# Epigenetic alterations of *gastrokine* 1 gene expression in gastric cancer

**DOI:** 10.18632/oncotarget.14817

**Published:** 2017-01-25

**Authors:** Filomena Altieri, Chiara Stella Di Stadio, Antonella Federico, Giuseppina Miselli, Maurizio De Palma, Emilia Rippa, Paolo Arcari

**Affiliations:** ^1^ Department of Molecular Medicine and Medical Biotechnology, University of Naples Federico II, Naples, Italy; ^2^ Hospital A. Cardarelli, Nasples, Italy; ^3^ CEINGE, Advanced Biotechnology Scarl, Naples, Italy

**Keywords:** gastrokine 1, gastric cancer, epigenetics, histone methylation, histone acetylation

## Abstract

The gastrokine 1 (GKN1) protein is important for maintaining the physiological function of the gastric mucosa. GKN1 is down-regulated in gastric tumor tissues and derived cell lines and its over-expression in gastric cancer cells induces apoptosis, suggesting a possible role for the protein as a tumor suppressor. However, the mechanism by which *GKN1* is inactivated in gastric cancer remains unknown. Here, we investigated the causes of *GKN1* silencing to determine if epigenetic mechanisms such as histonic modification could contribute to its down-regulation. To this end, chromatin immunoprecipitation assays for the trimethylation of histone 3 at lysine 9 (H3K9triMe) and its specific histone-lysine N-methyltransferase (SUV39H1) were performed on biopsies of normal and cancerous human gastric tissues. *GKN1* down-regulation in gastric cancer tissues was shown to be associated with high levels of H3K9triMe and with the recruitment of SUV39H1 to the *GKN1* promoter, suggesting the presence of an epigenetic transcriptional complex that negatively regulates *GKN1* expression in gastric tumors. The inhibition of histone deacetylases with trichostatin A was also shown to increase *GKN1* mRNA levels. Collectively, our results indicate that complex epigenetic machinery regulates *GKN1* expression at the transcriptional level, and likely at the translational level.

## INTRODUCTION

Gastrokine 1 (GKN1) is a tissue-specific 18 kDa protein that is highly expressed in the gastric mucosa of many mammalian species [1, 2]. Its biological function is poorly understood, but it is thought to be involved in the replenishment of the surface lumen epithelial cell layer, in maintaining mucosal integrity, and in cell proliferation and differentiation [3–6]. GKN1 is down-regulated in samples from *Helicobacter pyilori*-infected gastric mucosa but is absent in gastric adenocarcinoma tissues [1, 2, 7]. We previously showed that GKN1 down-regulation is one of the leading causes of gastric cancer (GC) development [8, 9]. Its over-expression in gastric adenocarcinoma cell lines AGS and MKN28 activated the expression of Fas receptor, while treatment with an anti-Fas antibody significantly increased apoptosis [10]. Moreover, treatment of tumor cells with recombinant human GKN1 reduced the proliferation of AGS cells compared with human embryonic kidney cells (HEK 293) and non-gastric cancer cells (H1355) [11]. These data suggest that GKN1 functions as a tumor suppressor and a modulator of apoptotic signals in GC. GKN1 could also be considered a biomarker for GC because individuals with a lower expression of the protein have an increased risk of developing gastric diseases [12].

*GKN1* (CA11, accession number: BK0017373) is located in a 6 kb region of chromosome 2p13 and contains six exons. The mechanism by which *GKN1* is silenced in GC and the role of epigenetic changes in this is unknown. Recently, Yoon *et al*. 2011 investigated this aspect in a sample group of 81 gastric carcinomas and 40 gastric adenomas [13]. No mutation was detected in gastric tumors and hyper-methylation of the *GKN1* promoter was only observed in two tumors, whereas DNA copy number and *GKN1* mRNA levels were significantly decreased in all GC samples. More recently, the Epstein–Barr nuclear antigen 1 (EBNA1) protein was reported to directly bind *GKN1* and *GKN2* promoters [14]. Treatment of AGS-Epstein–Barr virus (EBV) and AGS-EBNA1 cell lines with 5′ azacytidine showed that *GKN1* and *GKN2* were transcriptionally silenced by DNA methylation, and that latent EBV infection further reduced *GKN1* and *GKN2* expression in AGS cells. EBNA1 depletion by small interfering RNA partially alleviated this repression. However, the ectopic expression of EBNA1 slightly increased *GKN1* and *GKN2* basal mRNA levels, but reduced their responsiveness to demethylating agents. These findings indicated that EBNA1 contributes to the transcriptional complex and epigenetic deregulation of *GKN1* and *GKN2* tumor suppressor genes in EBV-positive GC.

Although these studies suggest that epigenetic modifications are involved in the deregulation of *GKN1* in GC, no studies have yet investigated histone modifications or the recruitment of histone-modifying enzymes and *GKN1* co-repressors in GC. Therefore, in the present study, we attempted to clarify whether epigenetic mechanisms are associated with *GKN1* silencing in GC and to determine whether this event might be involved in the development and progression of GC.

## RESULTS

### GKN1 expression levels in non-tumoral and tumoral tissues

We first analyzed the expression levels of GKN1 in six gastric tissue specimens from our collection of paired samples of non-tumoral (N_1_–N_6_) and tumoral (T_1_–T_6_) gastric tissues from the same patients. Tissue T_1_ showed a well-differentiated adenocarcinoma of intestinal type, T_2_ showed a severe dysplasia grade associated with a small area of intraglandular adenocarcinoma that was moderately differentiated, T_3_ and T_6_ showed a poorly differentiated adenocarcinoma of diffuse type, T_4_ showed a poorly differentiated adenocarcinoma of intestinal type, and T_5_ showed a moderately differentiated adenocarcinoma of intestinal type. The clinicopathologic characteristics of GC patients are summarized in Table 1.

**Table 1:** Characteristics of Gastric Cancer Patients

Variable	Gastric CancerSubjects (n = 6)
Age at surgery (Y)	
Mean	67 ± 13
Range	45 – 78
Sex ratio (M/F)	3/3
Tumor type	Intestinal 3, Diffuse 2, Dysplasia 1
Grade of differentiation	Well 1, Moderate 2, Poor 3

**Figure 1:**
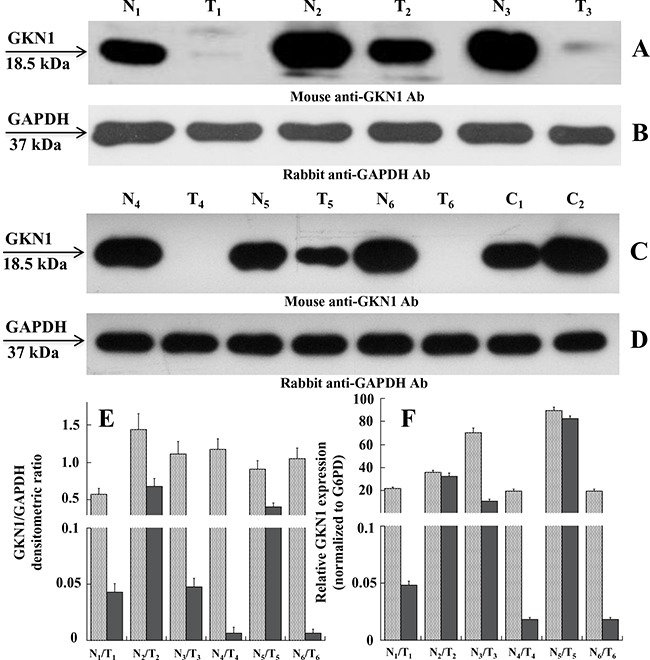
Expression levels of GKN1 in human gastric tissues **A** and **C**. Western blot of tissue extracts analyzed in paired non-tumoral (N_1_-N_6_) and tumoral (T_1_-T_6_) human gastric samples, respectively, using mouse anti-GKN1 antibody (Ab). **E**. Expression levels of GKN1 protein in non-tumoral (N_1_-N_6_) and tumoral (T_1_-T_6_) paired samples evaluated from the densitometry of GKN1 bands normalized towards the corresponding densitometry of GAPDH bands **B** and **D**. **F**. qRT-PCR analysis. Total RNA was prepared from gastric tissues and analyzed by qRT-PCR for *GKN1* mRNA level compared to *G6PD* mRNA as reference sample. Data from three experiments are reported as mean values ± SD.

The peritumoral areas of intestinal type GC showed a variable degree of gastric atrophy with diffuse intestinal metaplasia, while the peritumoral areas of diffuse type GC showed a variable degree of non-dysplastic inflammation. Figure 1 (panels A and C) shows the expression profiles of GKN1 in the six paired non-tumoral and tumoral tissues as evaluated by western blotting. Compared with non-tumoral tissues, all tumoral samples showed a down-regulation or an almost total absence of GKN1, using the GAPDH expression profile as a control (panels B and D) and based on the densitometric analysis of GKN1 expression (Figure 1E). Figure 1C shows the GKN1 expression profiles of samples C_1_ and C_2_ taken from healthy individuals undergoing sleeve gastrectomy as positive controls.

To determine if the observed down-regulation also occurred at the transcriptional level, we performed quantitative reverse transcription (qRT)-PCR on total RNA isolated from paired gastric non-tumoral and tumoral tissues. Figure 1F shows a decrease of *GKN1* mRNA levels in tumoral tissues compared with non-tumoral tissues, which supports the western blot findings.

### *GKN1* down-regulation in GC is associated with trimethylation of histone 3 at lysine 9 on the *GKN1* promoter

**Figure 2:**
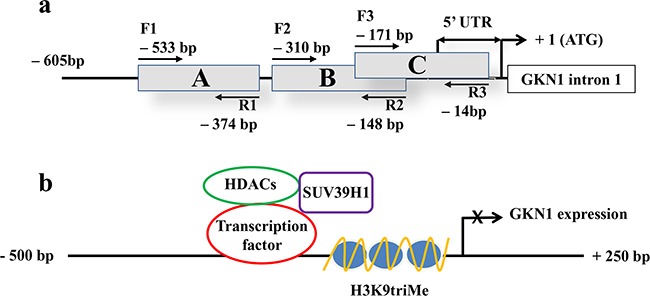
5′-region of *GKN1* gene analyzed by ChIP assay and proposed gene expression regulation **a**. The A, B and C regions of *GKN1* gene promoter delimited by the corresponding primer pairs (F1-R1, F2-R2, F3-R3) are boxed. The length of the 5′ UTR of *GKN1* mRNA is indicated by double arrows. +1 indicates the position of the start codon. **b**. Scheme showing the proposed mechanism of histone/SUV39H1 actions on *GKN1* gene promoter.

**Figure 3:**
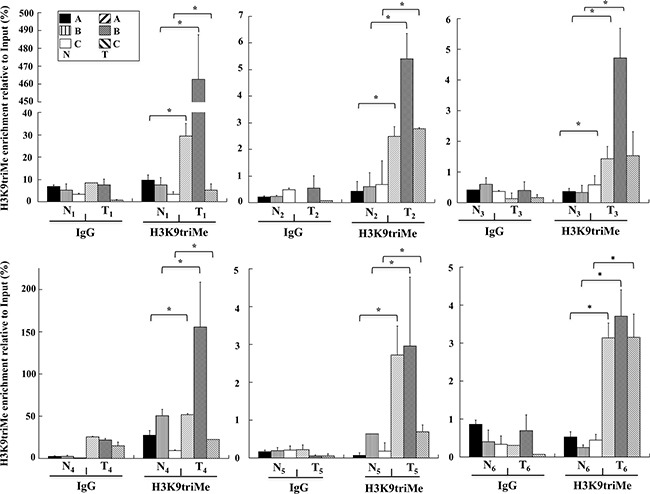
H3K9triMe levels on human *GKN1* gene promoter ChIP assays performed on human non-tumoral (N_1_-N_6_) and tumoral (T_1_-T_6_) human gastric samples, respectively. H3K9triMe enrichment relative to input is reported as 2^ΔCt^ × 100, where ΔCt is the difference between Ct_Input_ and Ct_IP_. All quantitative ChIP data were derived from three independent experiments, and for each experiment qPCR was performed in triplicate. * p<0.05, compared to corresponding control.

To investigate the possible causes of *GKN1* inactivation, chromatin immunoprecipitation (ChIP) assays for the repressive trimethylation of histone 3 at lysine 9 (H3K9triMe) were performed. A 600 bp promoter region of *GKN1* (identified by the UCSC Genome Browser) including the 5′-untranslated region (UTR) was divided into three different segments (A, B, and C) of about 160 bp, and corresponding PCR primers were designed (Figure 2A). ChIP assays performed on these three DNA segments revealed a significant increase in H3K9triMe modification in tumoral tissues compared with non-tumoral tissues. Figure 3 shows the results of an average of six independent experiments performed on six paired non-tumoral (N_1_–N_6_) and tumoral (T_1_–T_6_) specimens.

### H3K9triMe is associated with the recruitment and/or activation of a histone-lysine N-methyltransferase on the *GKN1* promoter

**Figure 4:**
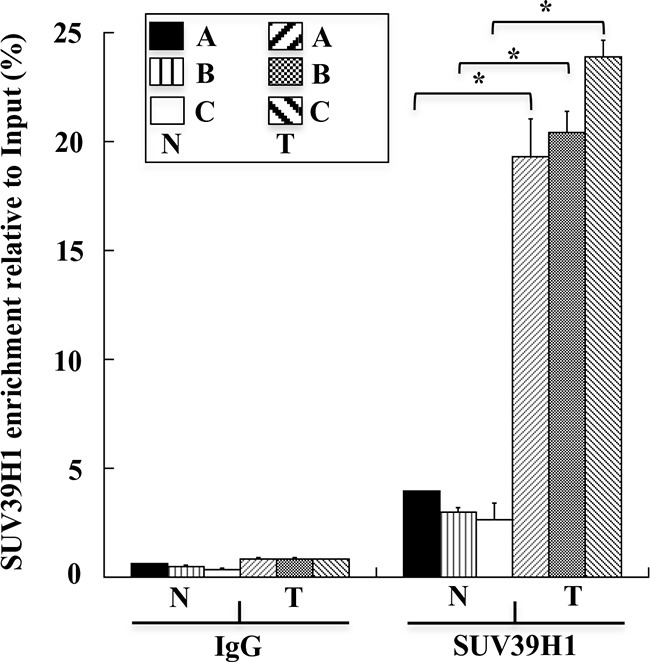
SUV39H1 levels on human *GKN1* gene promoter ChIP assays performed on human non-tumoral N_1_-N_6_(N) and tumoral T_1_-T_6_(T) human gastric samples, respectively. SUV39H1 enrichment relative to input is reported as 2^ΔCt^× 100, where ΔCt is the difference between Ct_Input_ and Ct_IP_. All quantitative ChIP data were derived from three independent experiments, and for each experiment qPCR was performed in triplicate. * p<0.05, compared to corresponding control.

Next, we examined the control of the H3K9triMe modification on the *GKN1* promoter. Previous work suggested that the H3K9triMe modification mainly occurs by a specific histone methyltransferase, histone-lysine N-methyltransferase SUV39H1, that trimethylates Lys-9 of histone H3 using mono-methylated H3 Lys-9 as a substrate [15]. Therefore, we used ChIP assays to verify the presence of this enzyme in the *GKN1* promoter region. Figure 4 clearly shows a significant increase in the binding of SUV39H1 to the *GKN1* promoter region in tumoral samples compared with non-tumoral tissues.

### The expression of SUV39H1, histone deacetylase 1, and H3K9triMe in GC tissues and cell lines

**Figure 5:**
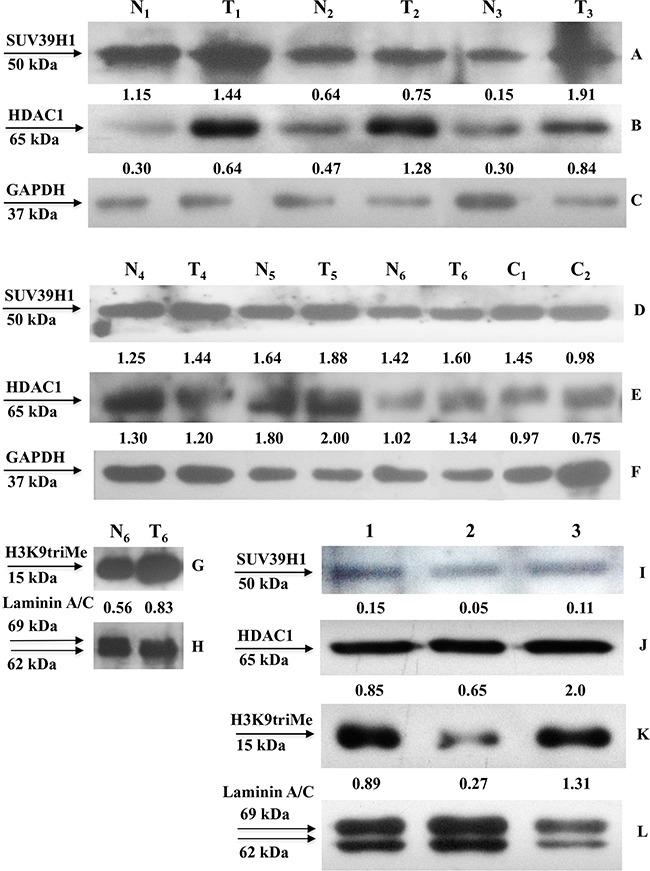
SUV39H1, HDAC1 and H3K9triMe expression in human gastric tissues and cell lines SUV39H1, HDAC1 expression in human non-tumoral (N) and tumoral (T) cell extracts (panels **A-F**) and H3K9triMe expression in nuclear extracts (panels **G-H**) was assessed by Western blot with the specific antibodies. Relative expression of SUV39H1, HDAC1 in sample tissues is reported as band intensity ratio with that of the corresponding GAPDH whereas that of H3K9triMe is shown as band intensity ratio with that of the corresponding lamin A/C. Protein expression in gastric cancer cell lines (panels **I-L**) was assessed by Western blot with the specific antibodies on nuclear extracts of AGS cells (lane 1), AGS cells transfected with flGKN1 (lane 2) and NCI-N87 cells (lane 3). Relative expression of SUV39H1, HDAC1 and H3K9triMe is reported as band intensity ratio with that of the corresponding lamin A/C.

We next investigated the relationship between the expression of GKN1 and that of SUV39H1, histone deacetylase 1 (HDAC1), and H3K9triMe. As shown in Figure 5 (panels A and D), we observed an increase in the expression of SUV39H1 in tumoral tissues (T_1_–T_6_) compared with non-tumoral tissues (N_1_–N_6_), based on the corresponding GAPDH western blot band intensity (Figure 5, panels C and F). Similar expression profiles for HDAC1 were seen in paired non-tumoral and tumoral gastric tissues (Figure 5, panels B and E). We were only able to analyze H3K9triMe expression in the nuclear extracts of one paired non-tumoral (N_6_) and tumoral (T_6_) sample. As shown in Figure 5G, tumoral tissue showed higher level of H3K9triMe expression compared with non-tumoral tissue, as determined by the western blot band intensity ratio with respect to that of lamin A/C (Figure 5H).

The expression levels of SUV39H1 and HDAC1 were next evaluated in healthy sleeve gastrectomy specimens (C_1_ and C_2_) (Figure 5, panels D and E). In these cases, expression levels appeared similar to those of non-tumoral tissues (N_4_–N_6_).

Lastly, we evaluated the expression levels of SUV39H1, HDAC1, and H3K9triMe proteins in GC cell lines. Because of the lack of a non-tumoral gastric cell line, we analyzed protein expression in GKN1-transfected and non-transfected GC cells (AGS) and in an additional non-transfected gastric cancer cell line (NCI-N87). As reported in Figure 5, AGS cells transfected with GKN1 demonstrated lower levels of SUV39H1 (Figure 5I), HDAC1 (Figure 5J), and H3K9triMe (Figure 5K) expression compared with non-transfected cells, as determined by the western blot band intensity ratio with respect to that of lamin A/C (Figure 5L).

### Treatment of GC cells with trichostatin A induces the up-regulation of *GKN1* mRNA

**Figure 6:**
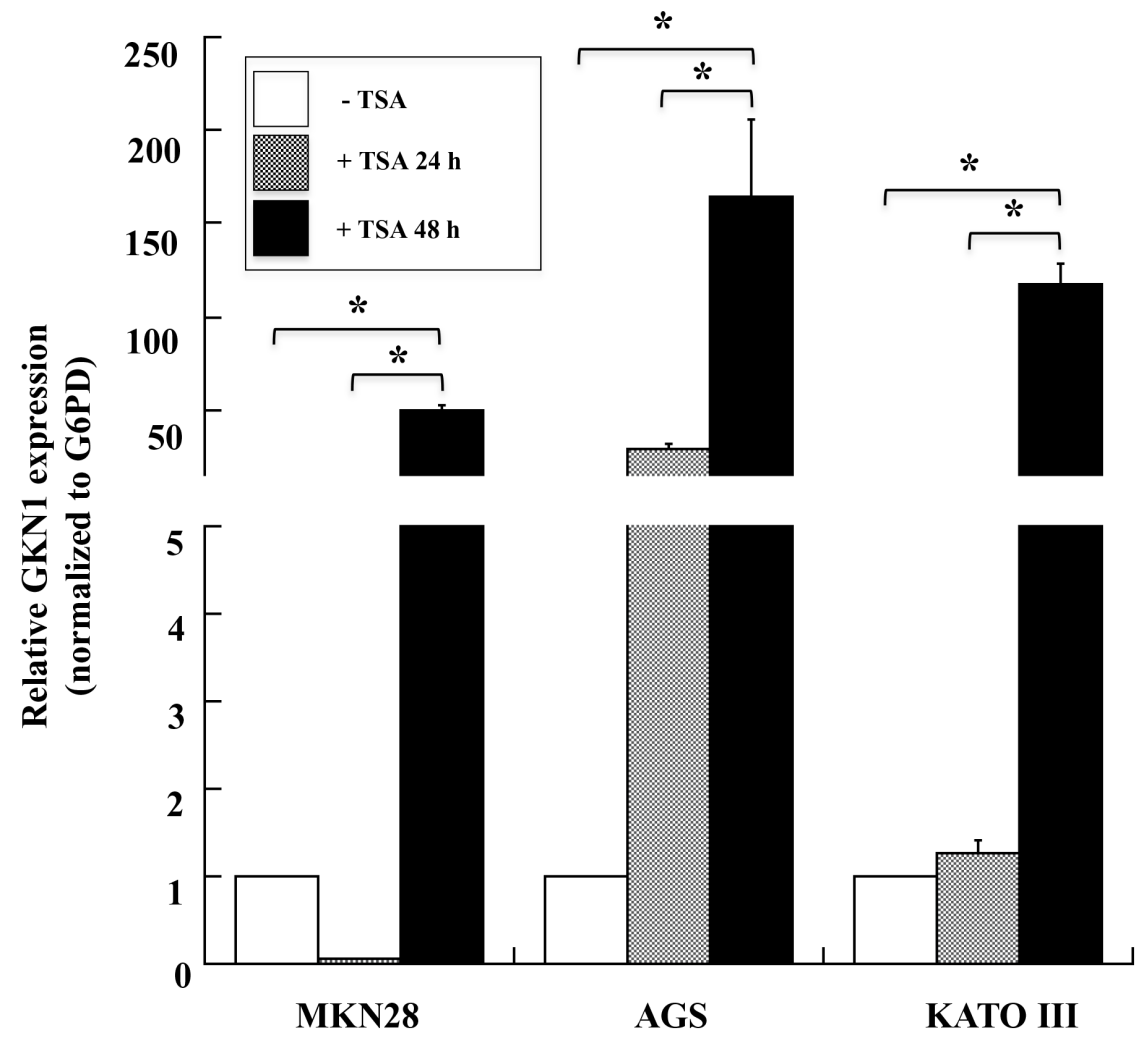
TSA induces the expression of *GKN1* mRNA in gastric cancer cell lines qRT-PCR analysis of *GKN1* mRNA in MKN28, AGS and KATO III gastric cancer cell lines after TSA treatment of the cells for 24 and 48 hours. *G6PD* was used as internal standard for normalization The relative expression of *GKN1* was evaluated using as control cells treated with DMSO. Data from a representative experiment are reported as mean values ± SD. * p<0.05.

To further understand the effect of epigenetic modifications on *GKN1* expression, we tested the possible role of histone acetylation. MKN28, AGS, and KATO III GC cell lines were treated with trichostatin A (TSA), an inhibitor of histone deacetylases (HDACs), and *GKN1* mRNA levels were evaluated by qRT-PCR. The treatment of AGS cells with TSA for 24 h led to an increase in *GKN1* mRNA expression of about 28-fold compared with untreated cells, whereas no effect was observed in MKN28 and KATO III cells. TSA treatment for 48 h increased *GKN1* mRNA expression by around 50-, 160- and 110-fold in MKN28, AGS, and KATO III cells, respectively (Figure 6). However, these results were not associated with protein re-expression, as evaluated by western blotting (data not shown).

**Figure 7:**
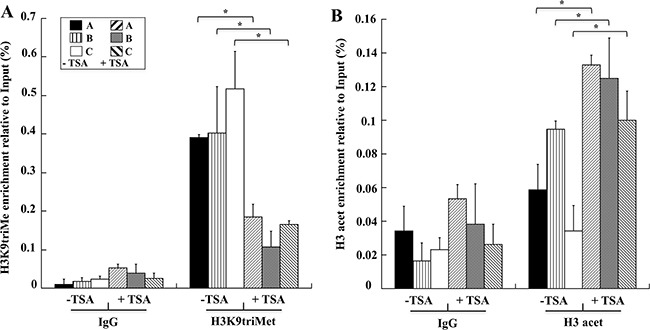
TSA induces in AGS cells decreased levels of H3K9triMe and increased levels of H3 acetylation Levels of H3K9triMe **A**. and H3 acetylation **B**. determined by ChIP assays on AGS cells not treated (-TSA) and treated with TSA (+TSA). H3K9triMe and H3 acetylation enrichment relative to input are reported as 2^ΔCt^ × 100, where ΔCt is the difference between Ct_Input_ and Ct_IP_. All quantitative ChIP data were derived from three independent experiments, and for each experiment, qRT-PCR was performed in triplicate. * p<0.05, compared to corresponding control.

The up-regulation of *GKN1* mRNA prompted us to use the TSA-treated AGS cell line as a positive control to confirm the results obtained in human gastric tissues in a cellular gastric model. As shown in Figure 7, a ChIP assay showed that *GKN1* up-regulation in AGS cells after TSA treatment was associated with a reduction of the H3K9triMe repressive modification (Figure 7A). This confirmed our findings in gastric tissues, and revealed an increase of H3-acetylation-activating modification in the same three regions of the *GKN1* promoter analyzed earlier (Figure 7B).

## DISCUSSION

Inflammation is a key risk factor in the development of many types of cancers, and chronic inflammation of the stomach initiates the histopathologic progression of chronic gastritis through gastric atrophy, intestinal metaplasia, dysplasia, and finally GC [16]. Indeed, *H. pylori* infection of the gastric mucosa and consequent chronic inflammation is a major step in the initiation and development of GC [17]. Promoter hypermethylation in tumor-related genes is often detected in premalignant gastric lesions [18], suggesting a relationship with the induction and or promotion of GC [19, 20]. Moreover, aberrant DNA methylation represents one of the most important inactivating mechanisms of tumor suppressor genes often associated with *H. pylori* infection [21, 22].

GKN1 expression is known to decrease throughout the progressive stages of neoplastic transformation. Previous investigations revealed no CpG hyper-methylation of the *GKN1* promoter in GC tissues [13] and altered GKN1 expression associated with the severity of gastritis and DNA methylation in non-neoplastic gastric mucosa [23]. Moreover, GKN1 expression in AGS cells induced endogenous micro RNA (miR)-185 that directly targeted the epigenetic effectors DNA (cytosine-5)-methyltransferase 1 (DNMT1) and histone-lysine N-methyltransferase enzyme (EZH2). The histone acetyl transferase Tip60 was shown to be up-regulated and HDAC1 to be down-regulated in an miR-185-independent manner, thus inducing cell cycle arrest by regulating cell cycle proteins in GC cells [24]. However, the precise relationship between gastritis and GKN1 has not been evaluated.

In the present study, we investigated the possible causes of *GKN1* inactivation by evaluating whether other epigenetic mechanisms could be involved in this process. Because DNA methylation, histone deacetylation, and methylation of histone H3 at lysine 9 are the three best-characterized covalent modifications associated with a repressed chromatin state, we focused our attention on these modifications [25, 26]. We show for the first time that a mechanism comprising histone modifications appears to be involved in the dysregulation of *GKN1* transcription in GC. By comparing human non-tumoral tissues with corresponding tumoral ones using ChIP assays, we revealed an increase of the repressive histone modification H3K9triMe (Figure 3) accomplished by recruitment of the specific histone methyltransferase SUV39H1 (Figure 4; Figure 5A, 5C) in tumoral tissues. Upregulation of SUV39H1 and H3K9triMe both at transcriptional and translational level has been demonstrated in several cancers therefore, SUV39H1 and H3K9triMe have important roles in cancer development and progression and the pharmacological inhibition of SUV39H1 may be a promising therapeutic approach for cancer treatment [27, 28]. Our present study documents the overexpression of Suv39H1 and histone tri-methylated H3K9 in gastric carcinoma.

It is interesting to note that although the enrichment levels of H3K9triMe modification observed in the six samples appeared quantitatively different, the *GKN1* promoter region where this modification was mostly enriched (region B) was the same in all samples. Additionally, no significant difference in H3K9triMe in non-tumoral gastric tissues was observed compared with tumoral ones, suggesting a correlation with the GKN1 protein expression shown in Figure 1. In fact, the H3K9triMe enrichment observed by ChIP (Figure 3) was associated with tumoral samples in which strong down-regulation of GKN1 expression was observed. The prominent role of H3K9triMe in GC was also confirmed by the finding that H3K9 trimethylation positively correlates with tumor stage, lymphovascular invasion, and cancer recurrence in gastric carcinoma whereas higher levels of H3K9 trimethylation correlate with poor survival [29].

Histone acetylation is one of the main determinants of chromatin structure [30], and it can be regulated dynamically through the involvement of transactivating factors with intrinsic histone acetylase activity or through the recruitment of deacetylase complexes that repress gene expression [31]. Indeed, several reports indicated that HDAC1 is up-regulated in many cancer cell lines and tissues [32], including GC, at both the transcriptional and translational levels [33]. Our findings are in agreement with this (Figure 5) and imply that increased HDAC1 expression causes histone hypoacetylation and the silencing of several tumor suppressor genes in GC. Therefore, we investigated whether underacetylation might contribute to *GKN1* transcriptional inhibition using TSA to increase general histone acetylation in an attempt to bypass the inhibitory effects of DNA methylation. Because TSA can arrest the cell cycle, induce apoptosis, regulate cell differentiation, and inhibit cell migration in the absence of cytotoxicity [34–36], we used milder experimental conditions (TSA, 90 ng/ml; time of treatment, 24 and 48 h) to reduce the possible inhibition of cell proliferation [37]. Under these same experimental conditions, TSA was previously shown to reduce AGS cell viability by less than 10% [38], and to dose-dependently inhibit MKN28 cell growth up to a concentration of 500 ng/ml [39]. Treatment of GC cell lines MKN28, AGS, and KATO III with TSA in the present study strongly increased *GKN1* mRNA expression (Figure 6), suggesting that histone deacetylation represents an important mediator of *GKN1* repression associated with DNA methylation. In fact, the TSA treatment of AGS cells led to a reduction of H3K9triMe and an increase of histone acetylation (Figure 6). Because histone acetylation is a fundamental regulatory mechanism for controlling gene accessibility, our results indicate that histone methylation is a unique mechanism for establishing local histone deacetylation, and generating maintainable epigenetic chromosomal states. However, it must be pointed out that even in this condition, the *GKN1* mRNA level was still very low because the cycle numbers required for its amplification were about 10-fold lower than those required for the amplification of housekeeping glucose-6-phosphate dehydrogenase (data not shown). In any case, no GKN1 protein re-expression was observed under these conditions by western blotting. This finding could be due to proteosome-mediated degradation of GKN1. To ascertain this possibility, we treated AGS cells with proteosome inhibitor (MG132). No GKN1 expression was observed (not shown). This suggests the presence of further regulation at the translational level, perhaps by mechanisms mediated by miRNAs, resulting in translational repression and gene silencing. For example, miRNA-544 directly targets the 3′-UTR of the newly-identified tumor suppressor gene *IRX1*, whose hypermethylation decreases expression of the protein in GC [40]. Therefore, miRNAs and promoter hypermethylation are important epigenetic mechanisms for transcriptional inactivation of tumor suppressors.

Recently, Yoon *et al*. 2015, showed that NKX6.3, considered a possible tumor suppressor for GC, is a transcriptional factor for *GKN1*. They showed that NKX6.3 is strongly down-regulated in GC cells; however, its over-expression in AGS and MKN1 GC cells induced the re-expression of GKN1 protein [41]. Therefore, it is possible that the strong increase in *GKN1* mRNA transcribed by over-expressed NKX6.3 in AGS cells escapes the post-transcriptional mechanism that regulates *GKN1* mRNA translation. With this in mind, the effects of GKN1 on SUV39H1, HDAC1, and H3K9triMe expression observed in transfected AGS cells in the present study (Figure 5) suggest that GKN1 functions as a direct or indirect modulator of the epigenetic factors involved in gene silencing during gastric carcinogenesis [24].

We propose the following model describing the mechanisms which H3K9triMe by SUV39H1 acts on the *GKN1* promotor (Figure 2B). A transcription factor functions as a negative regulator by recruiting SUV39H1 and HDACs to the *GKN1* promoter to induce histone deacetylation and methylation, thus resulting in *GKN1* repression. This model is in agreement with recent findings showing that restoration of GKN1 protein suppressed GC cell growth through an miRNA-mediated mechanism for DNA epigenetic modification [24]. Therefore, the loss of GKN1 function contributes to malignant transformation and the proliferation of gastric epithelial cells in gastric carcinogenesis.

## CONCLUSIONS

In conclusion, utilizing several specimens from patients with gastric carcinoma, we found that GKN1 expression is significantly reduced in dysplasia and tumor gastric mucosa, and is inversely correlated with the recruitment of H3K9triMe and Suv39H1 to the *GKN1* promoter. Tumoral GKN1 expression also appeared to be associated with an overall increase of the expression profiles of H3K9triMe, Suv39H1, and HDAC1 proteins. These findings provide evidence that epigenetic mechanisms leading to the inactivation of *GKN1* play a key role in the multi-step process of gastric carcinogenesis. While our results are relevant and reliable given that they were obtained *in vivo* using human specimens, we nevertheless aim to confirm them in a larger number of samples. Moreover, it will be also interesting to evaluate the role of miRNAs as regulators of GKN1 expression in GC. This will enable us to obtain a greater understanding of these mechanisms to determine whether they are involved in the development and progression of GC.

## MATERIALS AND METHODS

### Materials

Dulbecco’s modified Eagle’s medium (DMEM-F12) and fetal bovine serum (FBS) were purchased from Cambrex (Rutherford, NJ, USA). Mouse GKN1 monoclonal antibody (M01), clone 2E5, was purchased from Abnova (Taipei, Taiwan). Rabbit monoclonal to Histone H3 (tri methyl K9) [ab8898] and to HDAC1 [ab109411] antibodies were from Abcam (Cambridge, MA, USA), mouse SUV39H1 (clone MG44) [05–615] and rabbit acetyl-Histone H3 [06–599] antibodies were from Millipore (Temecula, CA, USA). DMSO and Trichostatin A (TSA) were from Sigma (Milan, Italy). Rabbit GAPDH monoclonal (glyceraldehyde-3-phosphate dehydrogenase) and rabbit Lamin A/C polyclonal antibodies were from Santa Cruz Biotecnology (Dallas, TX, USA).

### Cell cultures, transfection, human tissues and Western blotting

Human gastric adenocarcinoma cell lines (AGS, MKN28, KATO III, NCI-N87) were grown in DMEM-F12 supplemented with heat inactivated FBS, 1% penicillin/streptomycin and 1% L-glutamine at 37°C in a 5% CO_2_ atmosphere. AGS were transfected with 4 μg of vector pcDNA3.1-flGKN1(His)_6_ encoding the full length GKN1 (flGKN1, containing the first 20 amino acids leader peptide and His_6_-Tag sequence at the C-terminal) as already described [10]. The efficiency of transfection of gastric cancer cells with flGKN1 was always evaluated by a parallel transfection using EGFP vector as control. In general, after transfection, the average value of the ratio between number of green fluorescent cells/total number of cells was 0.5 ± 0.1.

Human gastric tissues were from patients with GC recruited at Hospital A. Cardarelli, Naples, Italy. All patients were interviewed regarding smoking habit, alcohol intake and chronic use of drugs. Hospital Pathologist performed the macro dissection of tumor and non-tumor tissues of GC patients during surgery. Gastric cancer was staged and graded according to the American Joint Committee on Cancer criteria [42]. The characterization of non-tumoral gastric mucosa was based on macroscopic aspects of normal compared with tumoral tissue as evaluated by the hospital pathologist [43], and from our previous work showing that GKN1 was highly expressed in gastric non-tumoral tissues but down-regulated or totally absent in GC tissues [3]. The study reported in the manuscript has been carried out in the frame of a research protocol entitled “Role of gastrokine 1 in gastric cancer” that has the approval from the Ethic Committee of the University of Naples Federico II (Comitato Etico Università Federico II). The assigned protocol number of the study was 34/15 [43].

Proteins from cell extracts (about 20 μg) were analyzed by Western blotting using mouse anti-GKN1 at 1:500, rabbit anti-Histone H3 (tri methyl K9) at 1:1000, rabbit anti-HDAC1 at 1:1000, mouse anti-SUV39H1 at 1:500, anti-GAPDH at 1:1000 and rabbit anti-Lamin A/C at 1:1000 dilution. Detection was performed using the enhanced chemiluminescence detection kit (SuperSignal West Pico) following manufacturer’s instructions. Western blot band intensity was measured with ImageJ 1.46r software.

### mRNA isolation and qRT-PCR

Total RNA was extracted from normal and cancer human tissues or from DMSO or TSA treated cells using TRIzol reagent solution (Invitrogen) according to the manufacturer’s protocol. cDNA was synthesized using the reverse transcription kit from Roche Molecular Systems (Roche, Penzberg, Germany) according to the manufacturer’s protocol. *GKN1* cDNA was amplified by qRT-PCR (forward and reverse primers 5′-ctttctagctcctgccctagc-3′ and 5′-tggttgcagcaaagccattt-3′, respectively) using the housekeepingglucose 6-phosphate dehydrogenase (G6PD) mRNA as an internal standard for normalization, according to standard procedures (Applied Biosystems, Foster City, CA, USA). qRT-PCR was performed with the SYBR Green PCR MasterMix (Applied Biosystems) under the following conditions: 10 minutes at 95°C, followed by 40 cycles (15 seconds at 95°C and 1 minute at 60°C). Each reaction was performed in triplicate. We used the 2^–ΔΔCT^ method to calculate the relative expression levels [44].

### Chromatin immunoprecipitation assay

Samples from normal and cancer human tissues were processed for chromatin immunoprecipitation (ChIP) assay. Cellular sospension was collected by centrifugation at 2000 rpm at 4°C for 10 minutes and then resuspended in 6× volume of cell lysis buffer [5 mM piperazine-N, N′-bis(2-ethanesulfonic acid) (PIPES) pH 8.0, 85 mM KCl, 0.5% NP-40] plus phenylmethylsulfonyl fluoride (PMSF) (1 mM) and trypsin inhibitor (10 μg/ mL) as protease inhibitors. Cells were then incubated on ice for 15 minutes and lysed using a dounce several times. Nuclei were collected at 5000 rpm at 4°C for 10 minutes and the pellet was resuspend in 5× volume of nuclei lysis buffer (50 mM TrisHCl pH 8.1, 10 mM EDTA, 1% SDS) plus the same protease inhibitors as the cell lysis buffer. The solution was incubate on ice for 20 minutes and subsequently freezed and thawed in liquid nitrogen 2 times to aid in nuclear lysis. After centrifugation at 5000 rpm at 4°C for 10 minutes, the obtained chromatin was sonicated according to the procedure described by Federico *et al*. 2009 [45]. Samples were subjected to IP with the following specific antibodies against histone modification anti-tri methyl K9-Histone3, anti-acetyl H3 and the specific histone methyltransferase anti-SUV39H1. For qRT-PCR, 2 μl aliquot of IP DNA (150 μl) were amplified with a set of three primers pairs (all primers are listed in the 5′ to 3′ direction); region A: F1, ggggtaggtttgg tgggagttgc, R1, atcacagctgaaaagccacgtgta; region B: F2, cgcccacagctttgactgggt, R2, tgccatgagccagtgtaccagga; region C: F3, tcctggtacactggctcatggca, R3, agcagtggacag aggagtaggca. GAPDH promoter amplicon was used as a negative control in all experiments (data not shown). IgGs were used as nonspecific controls, and input DNA values were used to normalize the values from quantitative ChIP samples.

ChIP assay from AGS cells treated with dimethyl sulfoxide (DMSO) or TSA were processed as above reported. For each assay, about 5×10^6^ AGS cells were used for chromatin preparation and IP.

### Treatment of gastric cancer cell lines with TSA

AGS, MKN28 and KATO III cells were plated in 10 cm culture dishes and grown for 24 hours before drug treatment. The next day, about 8.8 × 10^6^ cells were incubated in fresh culture medium containing a TSA/DMSO solution up to a final concentration of 300 nM. Control cells were treated with an equivalent volume of DMSO. After 24 or 48 hours, cells were harvested and used either to evaluate the *GKN1* mRNA expression by qRT-PCR or for ChIP assays.

### Statistical analysis

Statistical analysis was performed by two-tailed paired Student’s t-test using KaleidaGraph 4.1.1 software. Western blot band intensity was evaluated with ImageJ 1.41o software. Data were reported as means ± standard deviation (SD). The significance was accepted at the level of p < 0.05.

## Abbreviations

ChIP: chromatin immunoprecipitation
DMSO: dimethyl sulfoxide
DNMT1: DNA (cytosine-5)-methyltransferase 1
EZH2: enhancer of zeste homolog 2
GAPDH: glyceraldehyde-3-phosphate dehydrogenase
GC: gastric cancer
GKN1: gastrokine 1
G6PD: glucose-6-phosphate dehydrogenase
EBNA1: Epstein-Barr nuclear antigen 1
EBV: Epstein-Barr virus
HDAC: histone deacetylase
H3K9triMe: trimethylation of Histone 3 at lysine 9
IM: intestinal metaplasia
PIPES: 1,4-piperazinediethanesulfonic acid
PMSF: phenylmethylsulfonyl fluoride
Tip60: Histone acetyltransferase
TSA: trichostatin A
SUV39H1: Histone-lysine N-methyltransferase
